# The BSSO Foundry: A community of practice for ontologies in the behavioural and social sciences

**DOI:** 10.12688/wellcomeopenres.23230.1

**Published:** 2024-11-07

**Authors:** Janna Hastings, Lisa Zhang, Paulina Schenk, Robert West, Björn Gehrke, William R. Hogan, Bruce Chorpita, Marie Johnston, Marta M. Marques, Thomas L. Webb, Harriet M. Baird, Geert Crombez, Susan Michie

**Affiliations:** 1 Institute for Implementation Science in Health Care, Faculty of Medicine, University of Zurich, Zurich, Switzerland; 2 School of Medicine, University of St Gallen, St. Gallen, Switzerland; 3 Swiss Institute of Bioinformatics, Lausanne, Vaud, Switzerland; 4 Centre for Behaviour Change, University College London, London, England, UK; 5 Department of Behavioural Science and Health, University College London, London, England, UK; 6 Data Science Institute, Medical College of Wisconsin, Milwaukee, Wisconsin, USA; 7 Department of Psychology, University of California Los Angeles, Los Angeles, California, USA; 8 Institute of Applied Health Sciences, University of Aberdeen, Aberdeen, Scotland, UK; 9 National School of Public Health, Comprehensive Health Research Centre, Universidade NOVA de Lisboa, Lisbon, Portugal; 10 School of Psychology, The University of Sheffield, Sheffield, England, UK; 11 Department of Experimental-Health Psychology, Ghent University, Ghent, Flanders, Belgium

**Keywords:** ontology, behavioural and social sciences, community of practice, interoperable

## Abstract

There has been a rapid expansion in the quantity and complexity of data, information and knowledge created in the behavioural and social sciences, yet the field is not advancing understanding, practice or policy to the extent that the insights warrant. One challenge is that research often progresses in disciplinary silos and is reported using inconsistent and ambiguous terminology. This makes it difficult to integrate and aggregate findings to produce cumulative bodies of knowledge that can be translated to applied settings. Ontologies can address these challenges; their development and use have the potential to accelerate the behavioural and social sciences. Ontologies can facilitate communication through precise specification and dissemination of terms, and enable efficient data integration, sharing, comparison and analysis. The widespread use of ontologies in the biomedical and biological sciences has led to multiple successes. It is time now for the behavioural and social sciences to follow that lead.

In recent years, a number of ontologies have been developed within the behavioural and social sciences; however, efforts have tended to be isolated, with limited resources to support developers and those who work (or would like to work) with and use ontologies. There is a need for coordination and exchange to reduce duplication of work and leverage the value of a community to support the interoperability of these ontologies (linking of entities across domains and datasets). We have therefore initiated the Behavioural and Social Sciences Ontology (BSSO) Foundry, a community of practice and online repository for the development, adoption and use of ontologies in the behavioural and social sciences. The BSSO Foundry aligns with and builds upon the model provided by the Open Biological and Biomedical Ontology Foundry. We describe this new initiative and how to join and contribute to the community of interoperable ontologies for the behavioural and social sciences.

## Plain language summary

The behavioural and social sciences have produced a vast amount of research, yet the field faces substantial challenges. These include inconsistent and ambiguous use of terms across studies. Ontologies are structured frameworks to define terms and how they are related. They have been widely used in many areas of science, such as the biological and biomedical sciences. For example, the Gene Ontology describes and organises knowledge about the functions of genes across species. Recently, ontologies are being developed in the behavioural and social sciences to help systematically map and organise research plans and findings. For example, the Behaviour Change Intervention Ontology can be used to describe interventions that aim to change behaviour. However, to coordinate efforts, reduce duplication, and facilitate coherence and interoperability, we need a central community to enable exchange and support the use of ontologies. We have therefore set up the Behavioural and Social Sciences Ontology (BSSO) Foundry, a community of practice for the development, adoption, and use of ontologies within the behavioural and social sciences. We describe this initiative, including how to join and become involved.

## Disclaimer

The views expressed in this article are those of the author(s). Publication in Wellcome Open Research does not imply endorsement by Wellcome.

## Introduction

The behavioural and social sciences are essential to address global challenges, such as the climate and biodiversity crises, infectious and chronic diseases, antimicrobial resistance, food insecurity, and educational disparities, to name a few ( Bavel *et al.*, 2020; Hallsworth, 2023; Nielsen *et al.*, 2024). Behavioural and social sciences “study the complex interplay between biological, behavioral, social, and environmental processes, including phenomena that occur both within the organism (e.g., genetics, neurobiology, emotion, perception, cognition) and external to the organism (e.g., environment, social relationships, societal factors, culture, policy)” ( Office for Behavioral and Social Sciences Research [OBSSR], 2019). They include a broad and diverse array of disciplines such as, but not limited to, anthropology, economics, political science, psychology, human geography, and sociology.

The rapid expansion in the volume and complexity of data and evidence created in the behavioural and social sciences suggests potential, but also presents significant challenges. To illustrate, it has been estimated that over 100 papers reporting on trials of health behaviour change interventions are published every week ( West & Michie, 2023). Yet this proliferation of evidence is not informing scientific advance or policy sufficiently rapidly ( Glover *et al.*, 2018; Vroom & Massey, 2022). Reasons for this include working in disciplinary silos, and that the disciplines that contribute to the behavioural and social sciences often use different methods, vocabularies, definitions, and theories, making it difficult to aggregate and compare data and evidence ( Larsen *et al.*, 2017; Sharp *et al.*, 2023). Studies are also reported in highly varied ways, often with important information omitted, or inconsistent or ambiguous terminology used ( Michie *et al.*, 2009). For instance, “jingle-jangle” fallacies, where the same term is used for different phenomena, or different terms are used to describe to same phenomena, is a well-known problem that has hampered progress in the behavioural and social sciences ( Nigg *et al.*, 2002; Rothman & Sheeran, 2020). Not only does this lead to research waste ( Glasziou *et al.*, 2014), it also makes it difficult to synthesise evidence to produce cumulative knowledge that can be translated into practice and policy ( Sharp *et al.*, 2023). To advance the behavioural and social sciences, we need explicit and transparent conceptualisations and languages to link and integrate evidence across disciplines, research methods and topics.

Ontologies have been proposed as a method to meet this need by providing a structured, open and shared framework for clearly defining and specifying phenomena of interest (‘entities’) ( Larsen *et al.*, 2017; Michie *et al.*, 2022; Michie & Johnston, 2017) and the ways that they can be classified (‘classes’). For example, the Behaviour Change Technique Ontology ( Marques *et al.*, 2024) would describe an intervention that asked participants to do 30 minutes of physical activity five times a week as using the ‘set measurable behaviour goal BCT’ class. Ontologies are formal structures that represent phenomena within a domain in terms of uniquely specified classes of entities and relationships between them ( Hastings, 2017). An ontology provides a set of entities, each of which has (i) a unique identifier or ‘URI’ (e.g., BCIO:007300 in the example above), (ii) an unambiguous label and definition, and (iii) defined relationships with other entities ( Arp *et al.*, 2015). They may also contain additional metadata such as synonyms and cross-references ( Hastings, 2017).

Ontologies offer important benefits that can advance science. For example, they facilitate (i) the accumulation of knowledge by linking representations of entities across domains and data sets (termed ‘interoperability’), (ii) more efficient retrieval of information, integration and sharing of data, (iii) communication and collaboration across domains ( Sharp *et al.*, 2023; The Gene Ontology Consortium, 2015), and (iv) being explicit and transparent about conceptual definitions and assumptions. An influential report from the US National Academies of Sciences, Engineering, and Medicine (2022) recognised the importance of ontologies in advancing the behavioural and social sciences. One of the main conclusions was that ontologies have “the potential to move behavioral science forward from a domain in which research is generally siloed and the data and results are often incompatible to one in which the evidence is searchable and more easily integrated and in which computer technology is leveraged” (p. 5). In addition, the computational structure of ontologies allows them to be ‘read’ and processed by computers allowing researchers to harness the power of artificial intelligence approaches for automated reasoning and inference in large, complex datasets ( Hastings, 2017). Emerging methods are able to harness the logically structured knowledge from ontologies together with statistical approaches to artificial intelligence, such as language models, to enhance performance and support safer and more ‘grounded’ predictions ( Hastings, 2024).

The use of ontologies has become widespread in the biological and biomedical sciences; the Gene Ontology being one of the most widely used and successful examples ( Ashburner *et al.*, 2000). Recently, progress has been made with the development and adoption of ontologies in the behavioural and social sciences ( Baird *et al.*, 2023; Michie *et al.*, 2021). Several reviews ( Baird *et al.*, 2024; Blanch *et al.*, 2017; Braun *et al.*, 2023; Norris *et al.*, 2019) summarise efforts towards developing ontologies relevant to the behavioural and social sciences. In one example ( Baird *et al.*, 2024), 68 ontologies were developed that considered and conceptualized human behaviour, including ontologies designed to facilitate knowledge in the health, education, and legal domain. The review also extracted data (e.g., URIs, definitions, parent classes) relating to the concepts that are relevant to human behaviour, including concepts that describe how behaviors are measured (e.g., using self-report questionnaires, electronic devices, or biomedical markers) and described (e.g., who performs the behaviour or where the behaviour takes place). From the 68 ontologies identified for this review, 6079 concepts were extracted; 5449 of these concepts reflected behaviours, 251 reflected measures of behaviour, and 1382 reflected concepts that could be used to characterise behaviours. This points more broadly to a number of existing ontologies that may inform the development of ontologies in the behavioural and social sciences, including where ontologies can (and should) be integrated and aligned.

Recent investments in large-scale research programmes and initiatives (such as the US National Institutes of Health funding opportunity on the expansion of existing or development of new ontologies ^1^, and the Behavioural Research UK consortium which involves projects using ontologies ^2^) call for building a cumulative knowledge base in the behavioural and social sciences. However, comparatively few of the existing ontologies conform to the principles ^3^ of good ontology practice as set out by the Open Biological and Biomedical Ontology (OBO) Foundry ( Smith *et al.*, 2007). Examples of ontologies adhering to such principles include the Behaviour Change Intervention Ontology (BCIO) ( Michie *et al.*, 2021), the Addiction Ontology (ADDICTO) ( Hastings *et al.*, 2020), the Relationship Between Behaviours Ontology (RBBO) ( Mazumdar *et al.*, 2023), the Mental Health Ontology ( Schenk *et al.*, 2024a), and the Ontology for Modeling and Representation of Social Entities ( Hicks *et al.*, 2016). These ontologies have been created on the basis of shared principles, such as openness, collaboration, and best practices, including incorporating well-formed definitions for all terms in the ontology.

Ontologies in the behavioural and social sciences have begun to be applied in various ways. For example, the BCIO has been used to annotate study reports in evidence synthesis ( Norris *et al.*, 2024; West *et al.*, 2023), and to inform a novel machine learning algorithm for predicting smoking cessation outcomes ( Hastings *et al.*, 2023). Other work has focused on developing an ‘ontology-based modelling system’ to formally represent theories of behaviour change as triples of constructs and relationships ( Hale *et al.*, 2020; West *et al.*, 2019). These constructs can then be annotated, or mapped, to ontology classes for the purposes of searching, comparing and integrating theory ^4^. And the RBBO ontologies are being used as the basis for online tools that can be used to collate and integrate data on the relationship between behaviours ( Scott *et al.*, 2022). These applications show the potential benefits offered by ontologies in the behavioural sciences, including efficient integration of data and evidence, and integration of theories, both of which are important for cumulative science ( Hastings *et al.*, 2021).

## The benefits of a community and repositories for ontologies in the behavioural and social sciences

As the number of ontologies within the behavioural and social sciences grows, there is a need to easily locate ones that are relevant and when developing new ontologies to reuse relevant parts of existing ones (e.g., their classes and relationships) to avoid unnecessary overlap and reduce duplication of work. Dedicated repositories for this domain, separate from the pre-existing repositories in the biomedical domain, can help with this, especially if these resources also provide guidance about ontology development and standards and principles that are tailored to behavioural and social scientists (e.g., by using relevant examples). Using standards and principles is important because it allows ontologies to work together in an interoperable and coherent way ( Jackson *et al.*, 2021; Smith *et al.*, 2007). For example, the class ‘individual human behaviour’ (BCIO:036000) in the Human Behaviour Ontology ( Schenk *et al.*, 2024b) could be reused by ontologies related to physical activity (e.g. Carlier *et al.*, 2022), or ontologies for the relationships between behaviours (e.g., RBBO; Mazumdar *et al.*, 2023). This would enable a shared conceptualisation for ‘individual human behaviour’ which supports communication and integration of data about ‘individual human behaviour’ across ontologies. In addition, refinements made to classes in one ontology (e.g., additional synonyms added or further relationships specified between entities) can be easily adopted by another ontology that includes the same classes ( Masci *et al.*, 2009; Smith & Ceusters, 2010) without duplicating effort.

The behavioural and social sciences do not currently have a method for ensuring that ontologies are interoperable across different research teams, although work being conducted as part of Behavioural Research UK ^5^ – the DEMO-INTER project ^6^ – is developing and evaluating a workflow for enabling ontologies in the behavioural and social sciences to be interoperable. This work will be built on as part of the APRICOT (Advancing Prevention Research In Cancer through Ontology Tools) project, funded by the National Cancer Institute of the National Institutes of Health, which aims to further develop the BCIO for the domains of research methods, physical activity and smoking cessation as well as further develop ontology tools for the behavioural and social sciences community.

The OBO Foundry for ontologies in the biological and biomedical sciences provides a model of how to stimulate community development and exchange of an interoperable suite of ontologies. The OBO Foundry guides the development of ontologies according to common principles, enabling modular composition of ontologies and ensuring technical and scientific quality ( Smith *et al.*, 2007). For inclusion in the OBO Foundry, ontologies are required to follow a set of principles ^7^. There is now a growing appetite and a need for a similar but bespoke community for the behavioural and social sciences, recognising the unique aspects of this field and the topics it addresses that are differentiated from the already well-developed biomedical ontologies communities. These include systemic perspectives, emergent dynamics, human development, implementation aspects, alongside the increased complexity of integrating across the different disciplinary perspectives. Thus, we propose to address this need via the initiation of the Behavioural and Social Sciences Ontology (BSSO) Foundry. The BSSO Foundry will align with and build upon the OBO Foundry model and will facilitate linkage of participating ontologies to those in the OBO Foundry where relevant via the co-participation in both communities of some of the BSSO Foundry steering committee members (as detailed below) to ensure overall coherence.

## The BSSO Foundry

The BSSO Foundry provides a repository of ontologies, as well as an open community of practice and exchange (accessed at https://bssofoundry.org/). It aims to offer a central resource for guidance on the development, adoption and use of ontologies in the behavioural and social sciences. It will also act as a hub for collating and accessing tools and workflows that have been developed for researchers in the social and behavioural sciences to enable them to leverage the benefits of ontologies in their own work. While providing a central resource and infrastructure for exchanges, the community will encourage open dialogue across a broad range of stakeholders including actively seeking out participation and feedback from historically under-represented stakeholders.

The BSSO Foundry will serve to align ontology development efforts carried out by different research teams working within the behavioural and social sciences, fostering interoperability, and facilitating the re-use of classes where appropriate, while acknowledging the plurality of constructs in the domain ( Cornelius *et al.*, 2024) and allowing for the fact that ontologies can change and evolve over time. Its organisational structure is composed of (i) a steering committee to provide strategic and scientific guidance, and (ii) an operations committee to maintain the website and curate ontology metadata.

Members of the steering committee are world-leaders in the fields of behavioural and social science, computer science, and biomedical informatics (see Table 1). The steering committee members will be reviewed on an ongoing basis and community members who have participated actively for a significant period of time will be invited to apply to join the committee.

**Table 1.** Members of the Steering Committee of the BSSO Foundry (as of October 2024).

Bruce Chorpita	Professor of Clinical Psychology, UCLA, USA
Geert Crombez	Professor of Health Psychology, Ghent University, Belgium
Janna Hastings	Assistant Professor of Medical Knowledge and Decision Support, University of Zurich; co-participates in OBO Foundry community.
William R. Hogan	Professor and Director of the Data Science Institute, Medical College of Wisconsin, USA; co-participates in OBO Foundry community.
Marie Johnston	Emeritus Professor of Health Psychology, University of Aberdeen
Marta M. Marques	Assistant Professor for behavioural science and health promotion, NOVA University of Lisbon, Portugal
Susan Michie	Director of the Centre for Behaviour Change, University College London
Thomas L. Webb	Professor of Psychology, University of Sheffield, UK
Harriet Baird	Lecturer in Psychology, University of Sheffield, UK
Robert West	Professor Emeritus of Health Psychology, University College London

The lack of resources to support ontology developers was noted in the US National Academies of Sciences, Engineering, and Medicine’s report (2022). Therefore, the BSSO Foundry will seek to support the development and dissemination of tools and resources enabling the development and use of ontologies in the behavioural and social sciences (e.g., methods for matching ontologies and promoting interoperability, annotating datasets and so on). It will also play a role in organising and advertising wider education, training and dissemination efforts (e.g. workshops) that members can attend. Finally, through building a strong community around behavioural and social sciences ontologies, in the longer term the BSSO Foundry aims to advocate for the importance of ontologies in the behavioural and social sciences and take action to have impact on funding agencies for short-term and long-term funding.

The requirements for ontologies to join the BSSO Foundry include: (i) being within the scope of the behavioural and social sciences, (ii) conforming to principles of good ontology practice set out by the OBO Foundry, and (iii) a willingness for the authors of the respective ontologies to participate in community exchanges, coordination and knowledge transfer activities. The current active participating ontologies and their scope are summarised in Table 2. These ontologies have largely been developed using Basic Formal Ontology (BFO) as the upper-level organising structure ( Arp *et al.*, 2015).

**Table 2.** BSSO Foundry ontologies (as of September 2024).

Ontology	Scope	URL
Addiction Ontology (ADDICTO), including the E-Cigarette Ontology (E-CigO)	Addiction research and clinical practice	https://addictovocab.org/ ( Cox *et al.*, 2023; Hastings *et al.*, 2020)
Behaviour Change Intervention Ontology (BCIO)	Human behaviour change and behaviour change interventions	https://www.bciontology.org/ ( Michie *et al.*, 2021)
GALENOS mental health ontology (GMHO)	Mental health	https://www.galenos.org.uk/ontology ( Schenk *et al.*, 2024a)
Mental Functioning Ontology (MF)	Mental functioning	https://github.com/jannahastings/mental-functioning- ontology ( Hastings *et al.*, 2012)
Emotion Ontology (MFOEM)	Affective phenomena such as emotions, moods, appraisals and subjective feelings	https://github.com/jannahastings/emotion-ontology ( Hastings *et al.*, 2011)
Relationship Between Behaviours Ontology (RBBO)	Human behaviour and studies measuring relationships between behaviours	https://sites.google.com/sheffield.ac.uk/turbbo ( Mazumdar *et al.*, 2023)
Ontology for Modeling and Representation of Social Entities (OMRSE)	Human social interactions, such as social acts, social roles, social groups, and organizations.	https://github.com/mcwdsi/OMRSE/wiki/OMRSE-Overview ( Hicks *et al.*, 2016)
Contextualised and Personalised Physical activity and Exercise Recommendations (COPPER)	Support action and coping planning in the context of physical activity promotion by providing personalised recommendations for activities, activity context, barriers and coping strategies.	https://github.ugent.be/COPPER ( Braun *et al.*, 2024)

**Figure 1.**
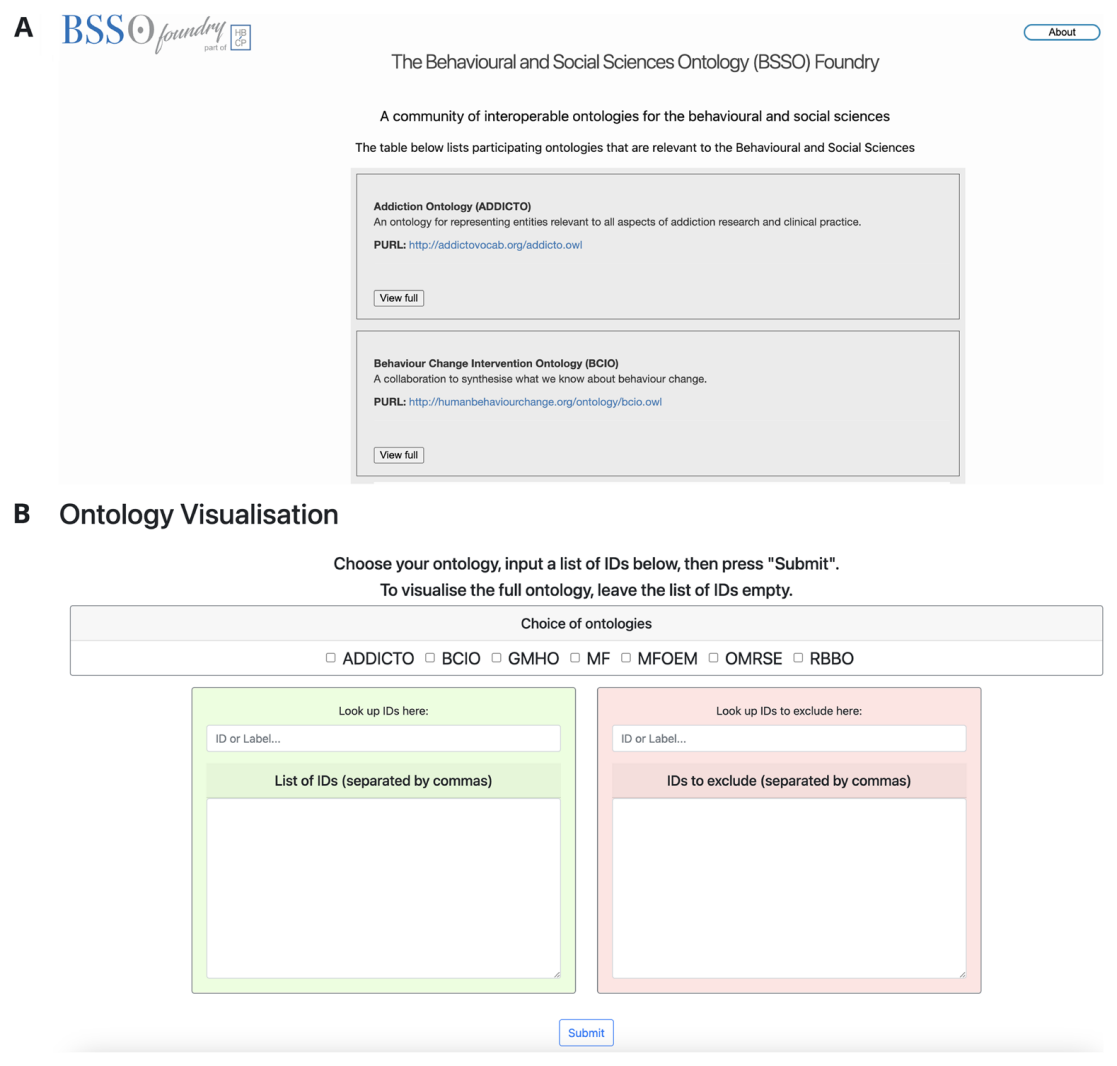
Screenshots of ( **A**) the BSSO Foundry website, which can be accessed at https://bssofoundry.org/, and ( **B**) the ontology visualisation tool, which can be accessed at https://vis.tools.bssofoundry.org/. The visualisation tool enables hierarchical visualisation of ontologies contained in the BSSO Foundry.

Joining the BSSO Foundry is free and offers the benefit of participation as well as access to Foundry resources. Initial resources offered by the BSSO Foundry in addition to the repository include a visualisation tool to diagrammatically represent entities within the BSSO Foundry ontologies ^8^. This tool offers network-based hierarchical visualisations of participating ontologies or selected portions thereof via an easy-to-use web-based interface ( Figure 1).

## Ways to get involved with the BSSO Foundry

The BSSO Foundry is an open community of practice; we actively invite any individual or group working in the domain of behavioural and social sciences to join. To participate and exchange with others in the community, we recommend joining the mailing list and discussion forum at http://groups.google.com/g/bssofoundry/.

Those who wish to submit a new ontology for inclusion in the Foundry should create an issue using the public issue tracker ( https://github.com/bssofoundry/bssofoundry.github.io/issues) with the description of the ontology, any relevant publications, and a link to the ontology OWL file. This will be considered by members of the steering committee on an ongoing basis who will assess the ontology against the requirements for joining (see above) and will support the submitters to address any actions required to ensure that the ontology is conformant with the principles and best practices, e.g. the use of standard identifier formats to support interoperability.

The issues tracker can also be used to report problems or request new features or activities.

## Conclusion

Ontologies have the potential to advance and accelerate the behavioural and social sciences. However, work is needed to enable behavioural and social scientists to adopt and actively use ontologies. The BSSO Foundry will serve as a new home for the growing community of ontology developers and users in the behavioural and social sciences. The Foundry will facilitate collaboration in developing, refining and maintaining ontologies – the overarching goal being to support transparent and explicit specifications of concepts, phenomena and ideas and a cumulative evidence base in behavioural and social sciences.

## Ethics and consent

Ethical approval and consent were not required.

## Data Availability

No data are associated with this article.

## Software availability

Source code available from: https://github.com/bssofoundry/ (The BSSO Foundry Repository) and https://github.com/ontology-tools/onto-vis (The BSSO Foundry visualisation tool)

Archived software available from: 10.5281/zenodo.13982866

License: CC-BY (Creative Commons Attribution 4.0 International)
