# Supplementary material for: The BSSO Foundry: A community of practice for ontologies in the behavioural and social sciences
Source: Wellcome Open Res. 2024 Nov 7;9:656. [Version 1] doi: 10.12688/wellcomeopenres.23230.1 (PMC11632217; doi:10.12688/wellcomeopenres.23230.1)
Supplement: Peer review history

# Reviewer report: Reviewer response for version 1

**DOI:** 10.21956/wellcomeopenres.25586.r111963

**Published:** 2024-12-11

**Authors:** Muhammad "Tuan" Amith

**Affiliations:** 1 The University of Texas Medical Branch Health, Galveston, Texas, USA

The authors of the manuscript introduces the Behavioral and Social Science Ontology (BSSO) Foundry, an ontology repository specific to ontologies representing social behavioral subdomains. The authors introduces the reader to a concise review of the landscape of social behavioral ontologies and the gap and need for them. It is presumed with the growth of data sets from diverse social behavioral datasets, there needs to be ontology models that can integrate and harmonize these datasets, as well as central repository as a "go to" source find ontologies. Leaning on the success of the OBO Foundry, the authors propose their own repository that replicates their practices and infrastructure. BSSO offers facilities to promote terminology reusability and interoperability, and has a multidisciplinary committee to shape the vision of the platform.

**Comments:**

- Overall the manuscript finely articulates background motivation for behavioral-social ontologies and would be a welcomed piece to share to those who are not familiar with the importance and role of ontologies for science research.
- Unfortunately the link to the repository (BSSO's main link) isn't active so I am unable to review the platform. Based on what is provided through the manuscript (visualization link), the visualization seem to be relatively simple compared to the features provided by BioPortal and OBO Foundry. In fact there is OntoPortal (https://ontoportal.org/) that provides the technology that is similar to BioPortal. Is there a rationale or maybe a comparison on how their own platform would differentiate from the others?
- I am not sure how the visualization can be helpful. After attempting to use it, I did get a simple tree structure but then again ontologies tend to be more complex and polyhierachal. I wonder if the platform should leverage VOWL to assist in the visualization or some pseudo-version of UML. It would be helpful to understand how a potential user might utilize the visualization. I also wonder if it might be useful to conduct some usability assessment with potential users?
- While I understand the need to have a central repository for behavioral and social-related ontologies, there is a concern that it might create silos. Maybe an explanation of why OBO or BioPortal may not be suitable to provide the support that is needed. Also term selection seems to be "localized" to the seven ontologies on the platform. How will the platform guarantee interoperability/coverage with terms from other repositories?

Does the article adequately reference differing views and opinions?

No

Are all factual statements correct, and are statements and arguments made adequately supported by citations?

Yes

Is the Open Letter written in accessible language?

Yes

Where applicable, are recommendations and next steps explained clearly for others to follow?

Yes

Is the rationale for the Open Letter provided in sufficient detail?

Yes

Reviewer Expertise:

knowledge engineering, public health informatics, semantic web, ontology, knowledge graph-driven software applications

I confirm that I have read this submission and believe that I have an appropriate level of expertise to confirm that it is of an acceptable scientific standard, however I have significant reservations, as outlined above.

# Reviewer report: Reviewer response for version 1

**DOI:** 10.21956/wellcomeopenres.25586.r111965

**Published:** 2024-12-10

**Authors:** Mark Musen

**Affiliations:** 1 Stanford University, Stanford, USA

This submission is not a scientific report but an announcement of the formation of a group of investigators who propose to serve as a steering committee for an informal organization to promote the use of ontology in the behavioral and social scientists and to ensure that ontologies in these disciplines adhere to certain unspecified standards.  The BSSO Foundry will be modeled on the OBO Foundry, which has had a significant effect on ontology development in biomedicine.

 The authors refer to the BSSO Foundry as a community of practice, but, as noted in the U.S. National Academies report that has stimulated recent evaluation of ontology development in the behavioral and social sciences, this is a discipline where currently there is neither much of a “community” nor much “practice.”  One does not create a community of practice simply by hanging up a shingle.  The OBO Foundry was successful because its formation followed nearly a decade of annual bio-ontology workshops at the ISMB conference and several meetings on “Standards and Ontologies in Functional Genomics” that nurtured a vibrant community.  The OBO Foundry also was kickstarted by significant funding from the U.S. National Institutes of Health, which was essential for consolidating the community and for developing infrastructure.  Yes, the OBO Foundry was announced with a publication, but its originators had to lay significant groundwork first.

 As noted by the authors, the U.S. National Institutes of Health launched an initiative to stimulate development of ontologies in the behavioral and social sciences.  This program supports a Behavioral and Social Science Ontology Coordinating Center and several individual grants to support practitioners who are building ontologies in this area.  One of the co-authors of the current paper is a recipient of one of these NIH grants for ontology development.  There is no discussion in the current paper how the BSSO Foundry initiative might relate to the newly funded BSSO program in the United States, and what synergies might accrue from collaboration.  It seems premature to publish an announcement of the BSSO Foundry without this discussion.  (Disclosure: I am a principal investigator of the BSSO Coordinating Center, and my colleagues and I eagerly seek collaboration.)

 The OBO Foundry has been in existence for nearly two decades.  What have the authors learned from observing the OBO Foundry experience?  What lessons might apply to the proposed BSSO Foundry initiative?  What will the authors do differently?  Why can’t the authors simply join forces with the OBO Foundry in some way?  Why can’t the BSSO Foundry take advantage of the enormous infrastructure for ontology development, dissemination, and use developed in the biomedical community?  These are all basic questions that need to be addressed by any organization that intends to pattern itself on the OBO Foundry and to lead the development of new terminological resources in a different area of science.

Does the article adequately reference differing views and opinions?

Partly

Are all factual statements correct, and are statements and arguments made adequately supported by citations?

Yes

Is the Open Letter written in accessible language?

Yes

Where applicable, are recommendations and next steps explained clearly for others to follow?

Yes

Is the rationale for the Open Letter provided in sufficient detail?

Partly

Reviewer Expertise:

Data science

I confirm that I have read this submission and believe that I have an appropriate level of expertise to confirm that it is of an acceptable scientific standard, however I have significant reservations, as outlined above.

# Reviewer report: Reviewer response for version 1

**DOI:** 10.21956/wellcomeopenres.25586.r111958

**Published:** 2024-11-30

**Authors:** Ray Stefancsik

**Affiliations:** 1 European Bioinformatics Institute (EMBL-EBI), Hinxton, UK

The authors present a compelling case for the creation of the Behavioural and Social Sciences Ontology (BSSO) Foundry, a comprehensive framework designed to advance research, analysis, and policy-making in the behavioural and social sciences. The manuscript effectively articulates the critical gaps in existing research infrastructure and demonstrates the potential benefits of this innovative collaborative platform.

 The paper builds robustly on the established principles of the Open Biological and Biomedical Ontology (OBO) Foundry. The BSSO Foundry's approach, characterized by modular ontology composition, ensures both technical and scientific rigor. Moreover, the transparent organizational structure and governance model are strategically designed to foster community engagement and knowledge exchange.

 I have verified that all web resources referenced in the paper are freely accessible and appear to be valuable tools for researchers. The abstract, main text, tables, and figures are clear, well-structured, and informative.

 One area for potential improvement is the "Plain language summary." While currently adequate, it could be enhanced to better communicate the concept to readers unfamiliar with ontologies. I recommend explicitly explaining that these ontologies are structured knowledge frameworks that use precisely defined terms and relationships comprehensible to both humans and computers. I think by explaining that this approach not only ensures interoperability but also provides scalability in data-intensive research areas would foster better understanding of the benefit the BSSO Foundry can provide and might facilitate wider community involvement.

 The manuscript successfully introduces the BSSO Foundry as a promising resource with significant potential to advance interdisciplinary research in the behavioural and social sciences.

Does the article adequately reference differing views and opinions?

Yes

Are all factual statements correct, and are statements and arguments made adequately supported by citations?

Yes

Is the Open Letter written in accessible language?

Yes

Where applicable, are recommendations and next steps explained clearly for others to follow?

Yes

Is the rationale for the Open Letter provided in sufficient detail?

Yes

Reviewer Expertise:

ontologies (information science); biomedical ontologies; biomedical databases; genetics; phenotypes; molecular biology

I confirm that I have read this submission and believe that I have an appropriate level of expertise to confirm that it is of an acceptable scientific standard.

# Reviewer report: Reviewer response for version 1

**DOI:** 10.21956/wellcomeopenres.25586.r110746

**Published:** 2024-11-26

**Authors:** Melissa Haendel

**Affiliations:** 1 The University of North Carolina at Chapel Hill, Chapel Hill, North Carolina, USA

This letter focuses on the effort to create and govern a suite of interoperable behavior and social ontologies. While it is true that standards for behavioral health and social sciences have been lacking and/or are not designed with interoperability in mind, it is not clear what scientific challenges the authors are trying to overcome with this new approach. Furthermore, they don't discuss existing standards and how they could be evolved or be interoperable with resources in the BSSO Foundry. This includes mental health standards such as DSM and RDOC, as well as other popular ontologies that contain behavioral terms, such as the Human Phenotype Ontology. 

 The manuscript would do well to better define the landscape and how the proposed BSSO ontologies fit within that landscape, as well as how they might advance specific scientific areas if the BSSO was successful. I would also recommend an evaluation of the content of the BSSO ontologies to illustrate how their interoperability is somehow more cohesive than the broader suite found outside the BSSO (OBO Foundry, clinical terminologies and standards, etc.). Finally, a set of use cases should be included to illustrate the kinds of data and process standardization that might be possible with the BSSO ontologies. The manuscript feels like a “build it and they will come” initiative without these use cases and more content evaluation.

**Minor:**

 The visualization tool figure should show content from one or more of the BSSO ontologies.

 Governance is not described, but there is a steering committee.

 The rationale for only including BFO-based ontologies is not clear. There are pros and cons to using BFO, and many users find BFO-based ontologies hard to adopt for a variety of reasons. BFO also has not always been adequate to represent complex entities with complex properties, which one might expect in the behavior and social domains.

Does the article adequately reference differing views and opinions?

Partly

Are all factual statements correct, and are statements and arguments made adequately supported by citations?

Yes

Is the Open Letter written in accessible language?

Yes

Where applicable, are recommendations and next steps explained clearly for others to follow?

No

Is the rationale for the Open Letter provided in sufficient detail?

Partly

Reviewer Expertise:

Genetics, semantic engineering, phenoptyping and precision medicine, clinical informatics

I confirm that I have read this submission and believe that I have an appropriate level of expertise to confirm that it is of an acceptable scientific standard, however I have significant reservations, as outlined above.
